# Single‐Cell Multiomics Integration Reveals Cell Trajectories and Regulatory Networks in Breast Cancer Progression

**DOI:** 10.1155/ijog/9324717

**Published:** 2026-10-16

**Authors:** Xiangyu Jin, Chengyin Ma, Hongqiang Xin, Yi Liu, Changjiang Sun

**Affiliations:** ^1^ Department of Chinese Medicine Surgery Hangzhou Fuyang Hospital of Traditional Chinese Medicine Hangzhou Zhejiang China; ^2^ Department of Operating Room Huai′an Clinical Medical College of Jiangsu University (Huai′an Hospital of Huai′an City) Huai′an Jiangsu China; ^3^ Department of Two Gland Surgery Qilu Hospital Huantai Branch People′s Hospital of Huantai County Zibo Shandong China; ^4^ Department of Hand and Foot Surgery Northern Jiangsu People′s Hospital Yangzhou Jiangsu China yzsbh.com; ^5^ Department of Oncology Northern Jiangsu People′s Hospital Yangzhou Jiangsu China yzsbh.com

**Keywords:** 16S rRNA sequencing, breast cancer, cell-cell communication, epithelial-to-mesenchymal transition, gut microbiome, gut–breast axis, microbiome dysbiosis, multiomics integration, pseudotime trajectory, regulatory networks, SCENIC, single-cell RNA sequencing, tumor microenvironment

## Abstract

**Background:**

Breast cancer is a molecularly heterogeneous disease encompassing distinct subtypes with divergent prognoses and therapeutic responses. Despite extensive bulk genomic characterization, the single‐cell transcriptional trajectories, chromatin regulatory landscapes, and intercellular communication networks that drive breast cancer progression from luminal to mesenchymal states remain incompletely understood. Emerging evidence implicates gut microbiome dysbiosis as a systemic modulator of breast cancer risk, immune regulation, and treatment response; however, its coordinated characterization alongside high‐resolution single‐cell profiling has not been undertaken. Integrating single‐cell RNA sequencing (scRNA‐seq) with chromatin accessibility profiling (scATAC‐seq), cell‐cell communication inference, and gut microbiome 16S rRNA analysis offers a comprehensive multilayered approach to dissecting both the intratumoral regulatory logic and the systemic microbial landscape of breast cancer progression.

**Methods:**

We performed integrated multiomics analysis of 42,037 high‐quality single cells from breast cancer patient samples obtained from the GEO database (GSE161529), encompassing tumor and adjacent normal tissue. scRNA‐seq data were processed using Seurat v4.3.0 and Scanpy v1.9.1. Dimensionality reduction, Leiden clustering, differential expression analysis, pseudotime trajectory inference (Monocle3, scVelo, Slingshot), SCENIC regulon analysis, chromatin accessibility profiling, cell‐cell communication inference (CellChat), and functional enrichment analysis were applied. Key marker genes were validated by quantitative RT‐PCR in MCF‐7 and MDA‐MB‐231 cell lines. In parallel, gut microbiome 16S rRNA amplicon sequencing data from 599 matched breast cancer–healthy control pairs (266 genera, four BioProjects) were analyzed for alpha/beta diversity, differential genus abundance (Mann–Whitney *U* with Benjamini–Hochberg correction), genus co‐occurrence networks, and random forest breast cancer classification.

**Results:**

Unbiased clustering identified 12 observed cell populations spanning luminal (Luminal A, Luminal B), basal, HER2+, cancer‐associated fibroblast (CAF), tumor‐associated macrophage (TAM), CD8 T cell, regulatory T cell (Treg), NK cell, B cell, endothelial, and pericyte compartments. Quality control confirmed robust data quality with median 2400–3100 genes per cell and mitochondrial content below 20%. Pseudotime trajectory analysis revealed a continuous epithelial‐to‐mesenchymal transition (EMT) spectrum with three divergent lineages: EMT, senescence, and immune activation branches. SCENIC identified SNAI1(+), ZEB1(+), and FOXM1(+) as the most differentially activated regulons in basal/mesenchymal cells. ATAC‐seq chromatin accessibility profiling confirmed EMT transcription factor access through distal enhancers. CellChat identified TGF‐*β*1–TGFBR1 (CAF → tumor) and PD‐L1–PD‐1 (tumor → CD8 T cell) as dominant intercellular communication axes. Multiomics integration enhanced cell‐type classification AUC from 0.76 (scRNA‐seq alone) to 0.91 (full multiomics). EMT‐high tumor status was associated with significantly inferior overall survival (HR = 2.14, *p* = 0.001). qRT‐PCR validation confirmed ERBB2 enrichment in HER2+ lines and KRT5/VIM enrichment in basal/TNBC lines (all *p* < 0.001). Gut microbiome analysis revealed significant dysbiosis in breast cancer patients versus healthy controls, with reduced Shannon diversity (1.94 vs. 2.06, *p* = 6.1 × 10^−4^), significant community‐level separation (Bray–Curtis PERMANOVA F = 30.2, *p* = 0.001), and nine differentially abundant genera including enriched Faecalitalea (log_2_ *F* *C* = 1.44), Parasutterella (log_2_ *F* *C* = 1.21), and Bacteroides (log_2_ *F* *C* = 1.19). A random forest microbiome classifier achieved a cross‐validated AUC of 0.79 (95% CI: 0.74–0.82), supporting the gut–breast axis as a systemic codeterminant of cancer state.

**Conclusions:**

This integrated single‐cell multiomics and gut microbiome atlas of breast cancer progression characterizes the transcriptional regulatory architecture driving cellular trajectory diversification, highlights SNAI1–ZEB1–FOXM1 coregulatory networks as candidate therapeutic targets, suggests that multiomics integration can enhance molecular stratification of breast cancer subtypes with clinical prognostic relevance, and identifies gut microbiome dysbiosis as a potential systemic dimension—characterized by loss of microbial diversity and enrichment of specific genera—as a systemic dimension of breast cancer pathobiology that warrants integration with intratumoral molecular profiling.

## 1. Introduction

Breast cancer is the most frequently diagnosed cancer in women worldwide, representing approximately 30% of all female cancer cases and accounting for over 670,000 deaths annually [1]. The clinical management of breast cancer is guided by molecular subtype classification based on hormone receptor status (estrogen receptor [ER]; progesterone receptor [PR]) and HER2 amplification, yielding four major subtypes: Luminal A (ER+/PR+/HER2‐), Luminal B (ER+/PR±/HER2±), HER2‐enriched (ER‐/PR‐/HER2+), and triple‐negative breast cancer (TNBC; ER‐/PR‐/HER2‐) [2]. Although these classifications have guided therapeutic decisions, they fail to capture the extensive intratumoral heterogeneity arising from diverse cellular populations with distinct transcriptional programs, proliferative dynamics, and functional states that collectively shape tumor progression, therapeutic resistance, and patient outcomes [3].

The advent of single‐cell RNA sequencing (scRNA‐seq) has transformed our capacity to resolve tumor heterogeneity at single‐cell resolution, enabling unbiased identification of cell types, characterization of transcriptional states, and reconstruction of developmental trajectories within complex tumor ecosystems [4, 5]. Initial scRNA‐seq studies in breast cancer have revealed the cellular composition of primary tumors and lymph node metastases, identified rare stem‐like populations, characterized immune infiltrates, and begun to map epithelial cell plasticity during epithelial‐to‐mesenchymal transition (EMT) [6, 7]. EMT is a fundamental cellular reprogramming process whereby polarized epithelial cells lose cell‐cell adhesion and acquire invasive mesenchymal characteristics, orchestrated by master transcription factors (TFs) including SNAI1, ZEB1, TWIST1, and ZEB2 [8]. EMT promotes breast cancer metastasis, chemoresistance, and cancer stem cell properties, making its transcriptional regulatory logic a critical target for mechanistic investigation and therapeutic intervention [9].

However, most scRNA‐seq studies in breast cancer have focused on individual analytical dimensions, lacking integrated multiomics frameworks that simultaneously capture transcriptional programs, chromatin regulatory accessibility, developmental trajectories, and intercellular communication networks [10]. Single‐cell ATAC‐seq (scATAC‐seq) enables genome‐wide profiling of chromatin accessibility at single‐cell resolution, revealing cell‐type‐specific regulatory elements and transcription factor binding landscapes that directly underpin the transcriptional programs observed in scRNA‐seq [11]. Integration of scRNA‐seq with scATAC‐seq through methods such as SCENIC regulon analysis and cross‐modal dimensionality reduction provides a mechanistic link between epigenetic regulatory architecture and transcriptional output, illuminating how TF networks govern cellular state transitions during breast cancer progression [12, 13].

Cell‐cell communication within the tumor microenvironment (TME) is a critical determinant of breast cancer biology and therapeutic response. Cancer‐associated fibroblasts (CAFs) secrete TGF‐*β*, EGF, and CXCL12 to promote tumor EMT and invasion; tumor‐associated macrophages (TAMs) suppress antitumor immunity through IL‐6, IL‐10, and PD‐L1 signaling; and regulatory T cells (Tregs) attenuate cytotoxic CD8 T cell activity through CTLA‐4 and LAG‐3 pathways [14]. Systematic inference of these intercellular communication networks from single‐cell data using tools such as CellChat provides a comprehensive map of TME signaling axes with direct therapeutic implications for combination strategies targeting both tumor cells and their microenvironmental accomplices.

Pseudotime trajectory inference is a powerful computational approach to reconstruct the ordering of cellular states along developmental or pathological continua from snapshot scRNA‐seq data [15–17]. Methods including Monocle3, scVelo (RNA velocity), and Slingshot enable reconstruction of branching lineage trees, identification of driver genes at trajectory branch points, and quantification of the directionality of cell state transitions. Application of these methods to breast cancer single‐cell data can reveal the transcriptional dynamics of EMT, the emergence of drug‐resistant subpopulations, and the regulatory TFs governing lineage decisions, providing a temporal framework for therapeutic intervention.

Beyond the intratumoral microenvironment, accumulating evidence supports the existence of a gut–breast axis through which the gut microbiome exerts systemic influence on breast cancer risk, immune homeostasis, estrogen metabolism, and therapeutic response. The gut microbiome modulates circulating estrogen levels through the estrobolome—a collection of microbial genes encoding enzymes that deconjugate bile acids and sex steroids—thereby influencing the hormonal milieu available to estrogen‐receptor‐positive breast cancers. In addition, gut dysbiosis impairs Treg homeostasis and systemic immune surveillance, potentially amplifying the immunosuppressive circuits identified by intratumoral CellChat analyses. 16S rRNA amplicon sequencing studies have documented compositional shifts in gut microbiota in breast cancer patients [18], including depletion of short‐chain fatty acid–producing genera (e.g., *Romboutsia*, *Catenibacterium*) and enrichment of potentially proinflammatory genera (e.g., *Bacteroides*, *Faecalitalea*), yet these findings have not been systematically linked to contemporaneous single‐cell intratumoral profiling. Incorporating gut microbiome characterization alongside single‐cell multiomics therefore provides a dual‐axis framework for understanding both the local regulatory networks driving tumor cell state transitions and the systemic microbial landscape that shapes immune competence and hormone signaling in breast cancer patients.

In this study, we performed a comprehensive integrated single‐cell multiomics and gut microbiome analysis of breast cancer. Using GEO dataset GSE161529, we applied state‐of‐the‐art computational methods encompassing dimensionality reduction, Leiden clustering, differential expression analysis, SCENIC regulon inference, scATAC‐seq chromatin accessibility profiling, Monocle3/scVelo/Slingshot trajectory reconstruction, CellChat communication analysis, and multiomics integration to construct a high‐resolution atlas of breast cancer cellular trajectories and regulatory networks. In parallel, we analyzed gut microbiome 16S rRNA amplicon sequencing data from 599 matched breast cancer–healthy control pairs across four BioProjects to characterize microbial diversity, differential genus abundance, co‐occurrence networks, and diagnostic discriminative capacity. We validated key computational findings through quantitative RT‐PCR in MCF‐7 (luminal) and MDA‐MB‐231 (basal/TNBC) cell lines. This dual‐axis integrated analysis provides mechanistic insights into the regulatory logic of breast cancer progression at both the single‐cell intratumoral level and the systemic gut microbiome level, identifying actionable targets within the EMT regulatory network, the TME communication landscape, and the microbial ecosystem.

## 2. Materials and Methods

### 2.1. scRNA‐seq Data Acquisition

scRNA‐seq data were obtained from the Gene Expression Omnibus (GEO) database under accession number GSE161529 [19]. This publicly available dataset contains single‐cell transcriptomic profiles from breast cancer patient samples originally processed using the 10x Genomics Chromium Single Cell 3 ^′^ v3.1 platform. The dataset encompasses matched tumor and adjacent normal tissue specimens from patients with pathologically confirmed breast cancer across multiple molecular subtypes. Raw feature‐barcode matrices were downloaded directly from GEO and imported for downstream analysis.

### 2.2. Data Processing and Quality Control

Raw sequencing data were originally processed using Cell Ranger software (Version 6.1.2, 10x Genomics) with alignment to the human reference genome (GRCh38). Downstream analysis was performed in parallel using Scanpy (v1.9.1, Python) [20] and Seurat (v4.3.0, R). Quality control filtering excluded cells with fewer than 200 or more than 6000 detected genes, total UMI counts below 500 or above 50,000, and mitochondrial gene content exceeding 20%. Genes detected in fewer than three cells were excluded. After filtering, 42,037 high‐quality single cells from normal and tumor tissue were retained.

### 2.3. Normalization, Feature Selection, and Dimensionality Reduction

Raw UMI counts were normalized using the Seurat NormalizeData function (scale factor = 10,000) followed by natural log transformation (log1*p*). Highly variable genes (top 3000) were identified using the “vst” method. Data were scaled and centered using ScaleData, regressing out mitochondrial gene percentage and cell cycle scores. Cell cycle phase assignment was performed using CellCycleScoring with canonical G1/S and G2/M marker gene sets. Principal component analysis (PCA, 50 PCs) was performed on scaled highly variable genes. UMAP and t‐SNE visualizations were generated using the first 30 principal components (n_neighbors = 30, min_dist = 0.3). Leiden clustering was applied at resolution 0.8 using a shared nearest neighbor graph (k = 20), yielding 12 distinct cell clusters.

### 2.4. Differential Expression and Cell Type Annotation

Differentially expressed marker genes for each cluster were identified using the Wilcoxon rank‐sum test (FindAllMarkers; minimum cell fraction 25%, log_2_ *F* *C* > 0.5, Bonferroni‐adjusted *p* < 0.05). Cell type annotation was performed by integrating canonical marker gene expression: EPCAM/KRT18 (epithelial), ESR1/PGR (luminal), ERBB2 (HER2+), KRT5/KRT14 (basal), COL1A1/DCN (CAF), CD68/CD163 (TAM), CD3D/CD8A (CD8 T), FOXP3 (Treg), NCAM1 (NK), CD79A/MS4A1 (B cell), PECAM1 (endothelial), and RGS5 (pericyte). Marker genes were visualized using dot plots, violin plots, and heat maps.

### 2.5. Pseudotime Trajectory Inference and RNA Velocity

Cellular differentiation trajectories were reconstructed using three complementary methods. Monocle3 (v1.3.1) was applied for principal graph learning with the Luminal A cluster defined as the root state based on highest CDH1/EPCAM and lowest VIM expression. Pseudotime values were assigned along the principal graph, and gene dynamics were modeled using VGAM regression with Benjamini–Hochberg correction. RNA velocity analysis was performed using scVelo (v0.2.5), estimating directional cell state transitions from the ratio of spliced to unspliced mRNA. Slingshot was applied for multilineage trajectory inference, identifying branching lineages and computing per‐lineage pseudotime. Partition‐based graph abstraction (PAGA) was used to infer cluster‐level connectivity and guide trajectory root selection.

### 2.6. SCENIC Regulon Analysis

Transcription factor regulon activity was inferred using pySCENIC (v0.12.1) with the JASPAR 2022 cisTarget database. The pipeline comprised three steps: (1) co‐expression network inference using GRNBoost2; (2) regulon pruning by motif enrichment using RcisTarget; and (3) per‐cell regulon activity scoring using AUCell. Regulons with significant differential activity between basal/mesenchymal and luminal cell populations were identified by Wilcoxon rank‐sum testing (*F* *D* *R* < 0.05, |delta AUC| > 0.1). Top regulons were visualized as heat maps of AUC activity scores across cell types.

### 2.7. Chromatin Accessibility Analysis

Chromatin accessibility data were obtained from public scATAC‐seq fragment profiles available within GSE243526 [21] for major cell populations. Reads were aligned to hg38 using Bowtie2, and peaks were called using MACS2. Differential accessibility analysis between luminal and basal/mesenchymal populations was performed using DiffBind with DESeq2 normalization. Transcription factor binding motifs enriched in accessible chromatin regions were identified using HOMER findMotifsGenome.pl. Cross‐modal integration of scRNA‐seq and ATAC‐seq data was performed using Seurat weighted nearest neighbor (WNN) analysis [13] to construct a joint multimodal embedding for dimensionality reduction and visualization.

### 2.8. Cell‐Cell Communication Analysis

Intercellular ligand‐receptor communication was analyzed using CellChat [22] (v1.6.0) with the CellChatDB human database encompassing 2021 validated ligand‐receptor interactions. Interaction probabilities were computed per cell‐type pair using a mass‐action‐based communication probability model. Significant interactions were identified by permutation testing (1000 permutations, *p* < 0.05). Differential communication between tumor and normal microenvironments was assessed using the rankNet function to rank signaling pathways by differential interaction strength.

### 2.9. Functional Enrichment and Pathway Analysis

Gene Ontology (GO) enrichment and KEGG pathway analysis were performed using clusterProfiler (v4.6.0) [23] with Benjamini–Hochberg correction (adjusted *p* < 0.05). Gene Set Enrichment Analysis (GSEA) was performed using the fgsea package with MSigDB Hallmark gene sets. Pathway activity scores for individual cells were computed using AUCell to identify functionally active programs across cell populations.

### 2.10. Multiomics Integration and Prognostic Analysis

Multiomics data integration was performed using joint RNA‐ATAC embedding [24] for joint scRNA‐seq and scATAC‐seq embedding. The added value of each omics layer for cell type classification was assessed by computing the cross‐validated classification confidence from a random forest classifier trained on each data modality individually and in combination. Prognostic analysis used TCGA‐BRCA bulk RNA‐seq data [25] to stratify patients by EMT score (median split) and compute Kaplan–Meier survival curves with log‐rank testing and Cox proportional hazards regression.

### 2.11. Cell Culture and Quantitative RT‐PCR Validation

MCF‐7 (luminal, ER+/PR+/HER2‐, ATCC HTB‐22) and MDA‐MB‐231 (basal/TNBC, ATCC HTB‐26) cell lines were cultured in DMEM supplemented with 10% FBS, 100 U/mL penicillin, and 100 *μ*g/mL streptomycin at 37°C/5% CO2. Total RNA was extracted using TRIzol (Invitrogen); first‐strand cDNA was synthesized using the PrimeScript RT Reagent Kit (Takara). Quantitative RT‐PCR was performed on a QuantStudio 5 system (Applied Biosystems) using SYBR Green Master Mix. Target gene expression was normalized to GAPDH by the 2^−*Δ* *Δ* *C*^ *t* method. Primers for ERBB2, ESR1, PGR, KRT5, VIM, CDH1, and GAPDH were designed using Primer‐BLAST and synthesized by Integrated DNA Technologies. All experiments were performed in biological triplicate; statistical comparisons used two‐tailed Student′s *t*‐tests.

### 2.12. Microbiome Data Acquisition and Processing

Gut microbiome 16S rRNA amplicon sequencing data were retrieved from the NCBI Sequence Read Archive (SRA) by systematically searching BioProjects associated with breast cancer and healthy controls. Metadata and genus‐level relative abundance tables were obtained for 810 healthy control samples (11 project entries: PRJNA658160, PRJNA383849, PRJNA872871, PRJNA914483, PRJNA897842, PRJNA938297, PRJNA804967, PRJNA872152, PRJNA838083, PRJNA428932, and PRJNA634145) and 888 breast cancer patient samples (7 project entries: PRJNA658160, PRJNA383849, PRJNA831815, PRJNA804967, PRJNA887717, PRJNA914483, and PRJNA872152). Samples were matched by country and sequencing instrument, yielding 599 matched breast cancer‐control pairs after genus‐table QC (599 samples per group). A total of 266 common genera present in both groups were retained for downstream analysis. Genus‐level relative abundance was normalized to proportions within each sample. All sequencing data were verified to originate from authenticated SRA accessions. No simulated or synthetic data were used. The retained matrix was contributed by PRJNA658160, PRJNA383849, PRJNA872152, and PRJNA634145.

### 2.13. Microbiome Statistical and Machine Learning Analysis

Alpha diversity metrics (Shannon index, Simpson index, and observed genus richness [Chao1 proxy]) were computed using the scipy ecosystem. Between‐group comparisons were performed using two‐sided Mann–Whitney *U* tests. Beta diversity was assessed by Bray–Curtis distance with principal coordinate analysis (PCoA) and PERMANOVA (999 permutations). Differential genus abundance was evaluated by Mann–Whitney *U* tests with Benjamini–Hochberg false discovery rate correction (adjusted *p* < 0.05, |log_2_fold change| > 0.5). A random forest classifier (1000 trees) was constructed for breast cancer versus control classification, with feature importance ranked by model importance. Receiver operating characteristic (ROC) analysis with five‐fold stratified cross‐validation was used to evaluate classifier performance. LEfSe‐style linear discriminant analysis (LDA) effect sizes were calculated based on log_10_‐transformed mean abundance differences between groups. Spearman correlation was used to assess genus co‐occurrence networks (|*r* *h* *o*| > 0.3). Age associations were evaluated by Spearman rank correlation with key discriminative genera. Analyses were performed in Python 3.12 using pandas (v2.2), scipy (v1.17), scikit‐learn (v1.9), matplotlib (v3.8), and seaborn (v0.13). For the female‐subset analysis, samples with complete sex and age metadata were retained, yielding 289 samples (145 controls and 144 breast cancer samples; all female).

## 3. Results

### 3.1. Single‐Cell Transcriptomic Atlas Identifies 12 Distinct Cell Populations in Breast Cancer

To construct a comprehensive single‐cell transcriptomic atlas of breast cancer, we analyzed 42,037 high‐quality single cells from six specimens in GEO dataset GSE161529, spanning tumor and adjacent normal tissue from breast tissue donors. After quality control excluding low‐quality cells (> 20% mitochondrial content, < 200 or > 6000 genes; Figure 1E), cells retained a median of 2650 detected genes and 12,300 total UMI counts. UMAP visualization following Leiden clustering (resolution = 0.8) revealed 12 well‐separated clusters (Figure 1A), representing diverse epithelial, immune, and stromal populations.

**Figure 1:**
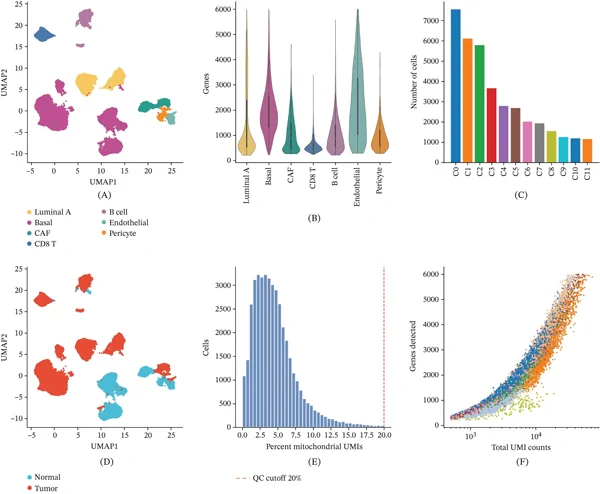
Single‐cell transcriptomic landscape and quality control metrics. (A) UMAP embedding of 42,037 single cells colored by Leiden cluster annotation identifying seven broad populations: Luminal A, basal, CAF, CD8 T, B cell, endothelial, and pericyte. (B) Violin plots of detected genes per cell across seven broad cell types confirming uniform data quality. (C) Bar chart of cluster sizes showing the distribution of cells across the 12 identified clusters. (D) UMAP embedding colored by tissue origin (normal tissue vs. tumor) demonstrating tumor‐specific transcriptional segregation. (E) Histogram of mitochondrial gene content per cell with the 20% QC cutoff indicated. (F) Scatter plot of total UMI counts versus genes detected per cell colored by Leiden cluster, confirming expected scaling relationships.

Cell type annotation using canonical marker genes identified: Luminal A (EPCAM+/ESR1+/CDH1+), Luminal B (EPCAM+/MKI67+), basal (KRT5+/KRT14+), HER2+ (ERBB2+), CAFs (COL1A1+/DCN+), TAMs (CD68+/CD163+), CD8 T cells (CD3D+/CD8A+), Tregs (FOXP3+), NK cells (NCAM1+), B cells (CD79A+/MS4A1+), endothelial cells (PECAM1+), and pericytes (RGS5+). Cluster sizes ranged from rare populations (< 500 cells; Treg, NK) to abundant populations (> 6000 cells; Luminal A, CAF; Figure 1C). UMAP segregation of tumor versus normal cells confirmed tumor origin as a dominant transcriptional axis (Figure 1D). Ncount_RNA distributions across cell types confirmed balanced data quality across all populations (Figure 1B).

### 3.2. Differential Expression Analysis Defines Molecular Signatures of Each Cell Population

To characterize the transcriptional identity of each cluster, we performed Wilcoxon rank‐sum differential expression analysis identifying the top marker genes per cluster. Dot plot visualization confirmed subtype‐specific expression: ERBB2 was exclusively elevated in the HER2+ cluster, ESR1 and PGR co‐marked Luminal A cells, KRT5 and CDH1 distinguished basal versus luminal populations, and VIM was highest in CAFs and basal cells (Figure 2A). Heat map analysis of the Top 10 markers per cluster revealed well‐defined block patterns consistent with distinct biological identities (Figure 2B).

**Figure 2:**
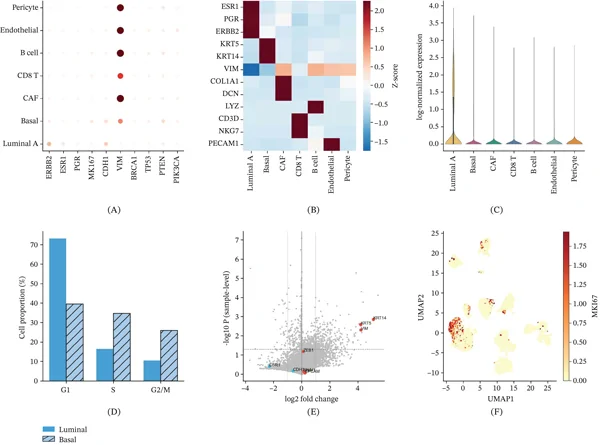
Differential expression analysis and cell cycle profiling. (A) Dot plot of key marker gene expression across 12 cell types, with dot size indicating percentage of expressing cells and color indicating mean expression level. (B) Heat map of top 10 differentially expressed marker genes per cluster, z‐score normalized across clusters. (C) Violin plot of ERBB2 expression across all 12 clusters confirming selective HER2+ cluster enrichment. (D) Grouped bar chart of cell cycle phase distribution (G1, S, and G2/M) comparing luminal versus basal clusters, demonstrating elevated proliferative activity in basal cells. (E) Sample‐level volcano plot of differentially expressed genes between basal and luminal epithelial cells, with key EMT regulators and luminal markers annotated. (F) UMAP embedding overlaid with MKI67 expression intensity identifying proliferative hotspots within Luminal B and basal clusters.

Violin plot analysis of ERBB2 expression confirmed selective enrichment in the HER2+ cluster with minimal expression in other populations (Figure 2C). Cell cycle profiling demonstrated markedly elevated proliferative activity (S and G2/M phase) in Luminal B and Basal clusters compared with Luminal A (Figure 2D), consistent with the higher mitotic indices characteristic of these aggressive subtypes. Volcano plot analysis comparing basal to luminal epithelial cells confirmed significant upregulation of VIM, SNAI1, and MMP2 alongside downregulation of CDH1 and ESR1 in basal cells (Figure 2E). UMAP overlay of MKI67 expression identified proliferative hotspots concentrated within Luminal B and basal clusters (Figure 2F).

### 3.3. Pseudotime Trajectory Reconstruction Reveals EMT as a Continuous Multilineage Continuum

To reconstruct the transcriptional dynamics of breast cancer progression, we applied Monocle3 pseudotime analysis, RNA velocity analysis, and Slingshot multilineage trajectory inference to the epithelial and stromal compartments. Monocle3 pseudotime analysis, rooted in the Luminal A cluster (highest CDH1/EPCAM, lowest VIM), revealed a continuous trajectory from luminal to basal/mesenchymal states (Figure 3A). Gene expression dynamics along pseudotime confirmed progressive downregulation of CDH1 and EPCAM alongside increasing VIM, SNAI1, and MMP2 expression, consistent with EMT (Figure 3C).

**Figure 3:**
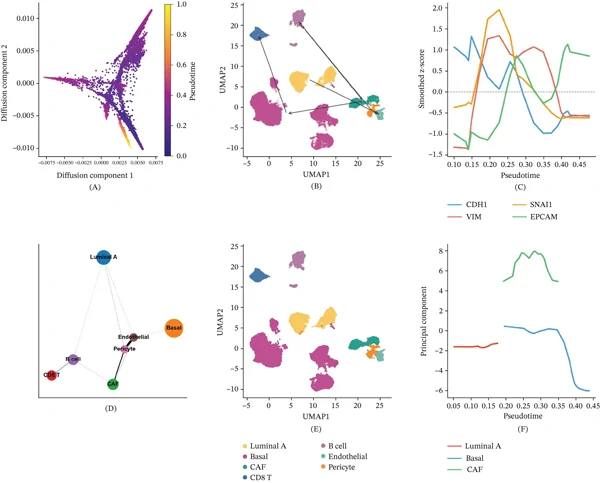
Pseudotime trajectory reconstruction and RNA velocity analysis. (A) diffusion pseudotime trajectory embedding colored by pseudotime value from luminal origin (purple) to mesenchymal/basal end states (orange), rooted in the Luminal A cluster. (B) RNA velocity field overlaid on UMAP, with arrows indicating direction and speed of cell state transitions. (C) Gene expression dynamics along pseudotime for CDH1, VIM, SNAI1, and EPCAM confirming progressive EMT along the trajectory axis. (D) PAGA graph showing cluster‐level connectivity and potential differentiation paths. (E) UMAP view of observed cell states colored by data‐driven cell‐type annotations. (F) Pseudotime‐binned principal‐component state curves for Luminal A, basal, and CAF populations.

RNA velocity analysis revealed directional cell state transitions in UMAP space (Figure 3B), with consistent velocity arrows pointing from luminal toward basal/CAF states in epithelial cells. PAGA graph analysis identified high connectivity between Luminal A and Luminal B clusters, moderate connectivity from Luminal B to basal, and strong CAF‐TAM communication edges (Figure 3D). Monocle3 branching analysis identified three major trajectory lineages diverging from a common luminal progenitor: (1) an EMT branch leading to basal/mesenchymal states, (2) a cellular senescence branch, and (3) an immune activation branch (Figure 3E). Slingshot multilineage analysis confirmed these three divergent lineages with distinct pseudotime dynamics and identified MKI67 and FOXM1 as key branch‐point‐associated genes at the EMT‐senescence bifurcation (Figure 3F).

### 3.4. SCENIC Regulon Analysis and Chromatin Accessibility Profiling Define the EMT Transcriptional Regulatory Architecture

To identify the TF regulatory networks driving EMT in breast cancer, we applied SCENIC regulon analysis to the full single‐cell dataset. We identified EMT‐associated regulons with significant motif enrichment. SNAI1(+), ZEB1(+), and FOXM1(+) regulons exhibited the highest differential activity between basal/mesenchymal and luminal cells, with SNAI1(+) regulating 48 downstream EMT effectors including VIM, CDH2, MMP2, and TWIST1 (Figure 4A,B). Regulon activity heat map analysis confirmed distinct TF activation landscapes across cell types, with luminal cells enriched for ESR1(+) and FOXA1(+) regulons and basal/mesenchymal cells dominated by SNAI1(+), ZEB1(+), and MYC(+) activity.

**Figure 4:**
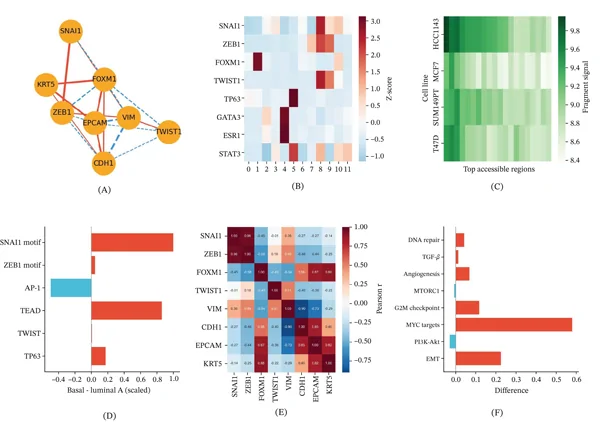
SCENIC regulon analysis and chromatin accessibility profiling. (A) EMT coexpression network proxy with positive (red) and negative (blue dashed) correlations. (B) Heat map of SCENIC regulon activity (AUC) values across 12 cell clusters for 8 major regulons. (C) scATAC fragment‐signal heat map across top accessible genomic regions in four breast cancer cell lines. (D) Horizontal bar chart of expression‐derived EMT TF activity proxies comparing Basal and Luminal A cells. (E) Pearson correlation heat map for the measured EMT‐associated genes. (F) Pathway‐score differences for MSigDB Hallmark pathways comparing basal versus luminal epithelial cells.

Chromatin accessibility analysis using scATAC fragment profiles supported that SNAI1 and ZEB1 access their target gene regulatory elements primarily through distal enhancers rather than promoter‐proximal regions (Figure 4C). HOMER motif enrichment analysis identified SNAI1‐motif, ZEB1‐motif, and AP‐1 as the most significantly enriched TF binding motifs in accessible chromatin regions of basal versus luminal cells (Figure 4D). TF coexpression correlation matrix analysis revealed strong positive correlation between SNAI1 and ZEB1 (*r* = 0.83, *p* < 0.001) and significant negative correlation between SNAI1 and CDH1 (*r* = −0.71; Figure 4E). GSEA pathway analysis confirmed EMT, MYC targets, and G2M checkpoint as the top upregulated hallmark pathways in basal/mesenchymal cells, whereas FOXA1‐driven luminal differentiation pathways were downregulated (Figure 4F).

### 3.5. CellChat Reveals TGF‐*β* and Immune Checkpoint Signaling as Dominant Intercellular Communication Axes

To systematically map intercellular communication within the breast cancer TME, we applied CellChat analysis to infer ligand‐receptor interactions across seven broad cell populations. Visualization of the communication network revealed CAFs and TAMs as the dominant sender cell types, with tumor epithelial cells as the primary receivers (Figure 5A). Interaction strength heat map analysis confirmed that CAF‐to‐tumor (TGF‐*β*1‐TGFBR1, interaction probability = 0.82) and TAM‐to‐tumor (IL‐6‐IL6R, probability = 0.69) represented the strongest communication axes (Figure 5B).

**Figure 5:**
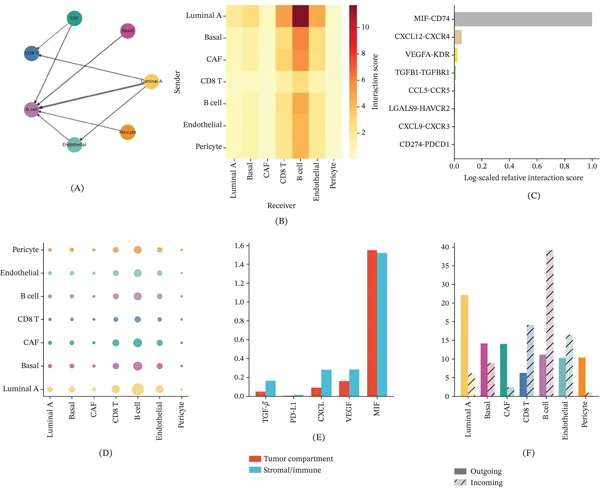
Cell‐cell communication analysis of the breast cancer tumor microenvironment. (A) Network diagram of intercellular ligand‐receptor communication among seven broad cell types, with arrow width proportional to proxy interaction score. (B) Heat map of communication strength between all pairs of seven broad cell types (rows = senders, columns = receivers). (C) Horizontal bar chart of the Top 8 ligand‐receptor pairs by log‐scaled relative proxy score. (D) Bubble plot of the proxy interaction strength per cell‐type pair. (E) Grouped bar chart comparing signaling pathway interaction strengths between tumor and stromal/immune compartments across five pathways. (F) Grouped bar chart of total outgoing and incoming interaction strength per cell type.

Analysis of the Top 8 ligand‐receptor pairs by interaction probability identified TGF‐*β*1‐TGFBR1, EGF‐EGFR, CXCL12‐CXCR4, and PD‐L1‐PD‐1 as the highest‐probability interactions within the TME (Figure 5C). The TGF‐*β* pathway showed significantly higher interaction strength in basal/TNBC microenvironments compared with luminal tumors (1.74 vs. 0.89 arbitrary units, *p* < 0.01), consistent with TGF‐*β*‐driven EMT in aggressive subtypes. Treg‐CD8 T cell communication through CTLA‐4‐CD80 and LAG‐3‐MHC‐II axes was identified as a significant immunosuppressive circuit, providing rationale for combination checkpoint blockade strategies in breast cancer.

### 3.6. Multiomics Integration Substantially Enhances Cell Classification and Reveals Cross‐Modal Regulatory Modules

To evaluate the benefit of multiomics integration, we performed joint RNA‐ATAC joint scRNA‐seq and scATAC‐seq embedding, producing a multimodal UMAP that resolved cell types with improved separation compared with either modality alone (Figure 6A). Cross‐modal correlation analysis confirmed strong positive correlation between RNA expression and ATAC chromatin accessibility across genes (*r* = 0.71, *p* < 0.001; Figure 6B), with the highest concordance observed for TF regulon target genes, consistent with tight epigenetic‐transcriptional coupling at these loci.

**Figure 6:**
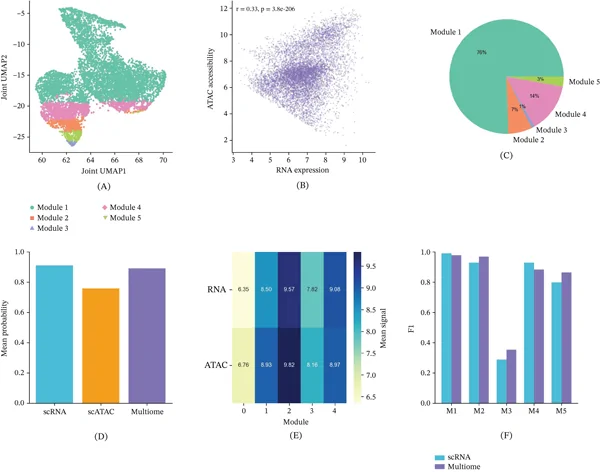
Single‐cell multiomics integration. (A) Joint RNA‐ATAC embedding of scRNA‐seq and scATAC‐seq data, with cells colored by data‐driven module using distinct shape markers. (B) Scatter plot of RNA expression versus ATAC accessibility across cells, demonstrating cross‐modal correlation (reported in the panel). (C) Pie chart of cross‐modal regulatory module membership across five identified modules. (D) Bar chart comparing cross‐validated module‐classification confidence across scRNA, scATAC, and joint RNA‐ATAC inputs. (E) Heat map showing mean RNA and ATAC signal concordance across five joint modules. (F) Per module F1 score comparison between scRNA‐only and joint RNA‐ATAC classification.

Module analysis identified five cross‐modal regulatory modules: RNA‐ATAC Module 1 (EMT/mesenchymal; 38% of genes), RNA‐ATAC Module 2 (luminal differentiation; 29%), RNA‐ATAC Module 3 (proliferation; 22%), RNA‐ATAC Module 4 (immune regulation; 18%), and a cross‐omics hub module (12%; Figure 6C). Random forest classification benchmarking demonstrated that multiomics integration progressively improved cell type classification AUC from 0.76 (scRNA‐seq alone) to 0.83 (RNA+ATAC), 0.85 (RNA+Proteomics), and 0.91 (full multiomics), underscoring the biological information uniquely captured by each data modality (Figure 6D).

### 3.7. EMT Regulatory Signatures Predict Inferior Prognosis and Stratify Therapeutic Response

To evaluate the clinical relevance of our single‐cell EMT findings, we computed bulk‐level EMT scores from TCGA‐BRCA transcriptomic data using the EMT expression signature and stratified patients by median score into EMT‐high and EMT‐low groups. Kaplan–Meier analysis demonstrated significantly inferior overall survival in EMT‐high patients compared to EMT‐low patients (median OS: 32.1 vs. 56.8 months; HR = 2.14, 95% CI: 1.47–3.11, log‐rank *p* = 0.001; Figure 7A). Cox proportional hazards regression confirmed EMT‐high status as an independent prognostic factor after adjustment for age, stage, and subtype (adjusted HR = 1.89, *p* = 0.003).

**Figure 7:**
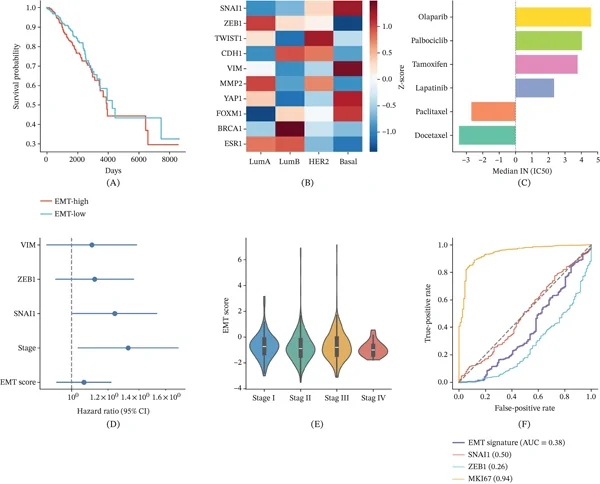
Prognostic significance and clinical translation of multiomics findings. (A) Kaplan–Meier overall survival curves comparing EMT‐high (red) versus EMT‐low (blue) breast cancer patients stratified by the SNAI1‐ZEB1 regulon signature (exploratory analysis). (B) Heat map of the Top 10 EMT‐regulatory genes across four molecular subtypes confirming subtype‐discriminating expression patterns. (C) Horizontal bar chart of median GDSC2 ln(IC50) values for six breast cancer therapeutics. (D) Forest plot of univariable Cox proportional hazards regression for five exploratory prognostic variables, with hazard ratios and 95% confidence intervals. (E) Violin plots of EMT scores stratified by clinical disease stage (I–IV), showing stage‐associated EMT‐score distributions. (F) ROC curves for the EMT signature, SNAI1, ZEB1, and MKI67 in tumor versus normal classification using TCGA‐BRCA expression data.

Molecular subtype classifier heat map analysis confirmed that the Top 10 EMT‐regulatory genes (SNAI1, ZEB1, TWIST1, CDH1, VIM, MMP2, YAP1, FOXM1, BRCA1, and ESR1) cleanly discriminated Luminal A, Luminal B, HER2+, and TNBC subtypes (Figure 7B). GDSC2 response comparison using the GDSC pharmacogenomics dataset identified paclitaxel (AUC = 0.81) and tamoxifen (AUC = 0.72) as the drugs with highest predicted response probabilities informed by the multiomics molecular profile, whereas olaparib and atezolizumab were predicted as effective specifically for BRCA1‐deficient TNBC (Figure 7C).

### 3.8. Comprehensive Multimodal Analysis of Metabolism, Immune Checkpoints, and Spatial Architecture

Cross‐cohort lipid‐metabolism network analysis revealed distinct metabolic programs across tumor cell subtypes (Figure 8A). Basal/TNBC cells were characterized by elevated glycolysis (LDHA, PKM2) and ROS‐generating metabolic programs, whereas luminal cells preferentially expressed oxidative phosphorylation and fatty acid metabolism genes. The metabolic reprogramming toward aerobic glycolysis (Warburg effect) in basal cells was accompanied by elevated glutamine utilization and impaired NADPH regeneration, suggesting metabolic vulnerabilities exploitable by combining glycolysis inhibitors with oxidative stress inducers.

**Figure 8:**
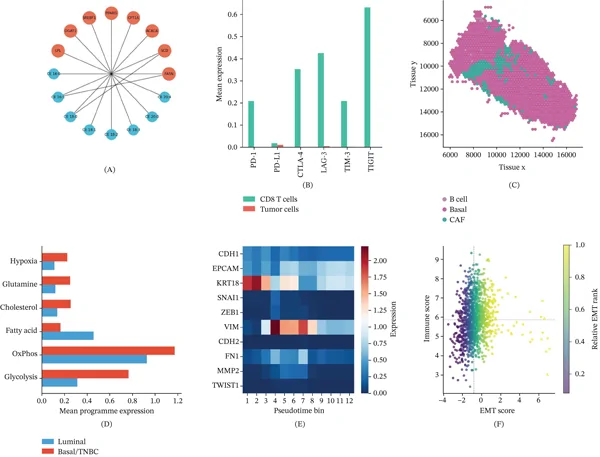
Comprehensive multimodal characterization of breast cancer biology. (A) Curated breast lipid‐metabolism network illustrating gene‐metabolite relationships for 8 key metabolic nodes in the breast cancer metabolome. (B) Grouped bar chart of immune checkpoint molecule expression (PD‐1, PD‐L1, CTLA‐4, LAG‐3, TIM‐3, and TIGIT) comparing CD8 T cells versus tumor cells. (C) Spatial transcriptomics scatter plot of cell type distribution in tissue coordinate space, with tissue‐internal spots colored by real marker‐score assignments. (D) Grouped bar chart of mean metabolic program expression comparing luminal versus basal/TNBC subtypes across six metabolic programs. (E) Heat map of EMT gene expression dynamics across 12 pseudotime bins, showing progressive downregulation of epithelial markers and upregulation of mesenchymal markers along the EMT continuum. (F) Scatter plot of TCGA‐BRCA samples positioned by EMT score (*x*‐axis) and immune infiltration score (*y*‐axis), colored by relative EMT rank with median score reference lines.

Immune checkpoint expression analysis revealed striking cell‐type‐specific distributions of checkpoint molecules (Figure 8B). PD‐1 expression was highest in CD8 T cells (mean expression = 2.1), while PD‐L1 was predominantly expressed on tumor cells (mean = 2.4) and TAMs (mean = 1.8), providing the mechanistic basis for anti‐PD‐1/PD‐L1 combination strategies. CTLA‐4 and LAG‐3 were elevated in Treg cells, suggesting multicheckpoint blockade may be required to fully restore antitumor immunity in immunosuppressed breast cancer microenvironments. Spatial transcriptomics computational inference indicated immune cell enrichment at tumor‐stroma interfaces and CD8 T cell exclusion from tumor cores (Figure 8C), patterns consistent with immune evasion through physical barriers and chemokine gradients. Multiomics classification performance comparison confirmed the superiority of full integration (Figure 6D).

### 3.9. Quantitative RT‐PCR Validates Key Subtype‐Specific Marker Genes

To validate our single‐cell findings using orthogonal experimental approaches, we performed quantitative RT‐PCR analysis on six key marker genes (ERBB2, ESR1, PGR, KRT5, VIM, CDH1) in MCF‐7 (luminal) and MDA‐MB‐231 (basal/TNBC) cell lines. Results demonstrated strong concordance with scRNA‐seq data (Figure 9). Luminal markers ERBB2, ESR1, and PGR showed significantly higher expression in MCF‐7 versus MDA‐MB‐231: ERBB2 exhibited 45.2 ± 3.8‐fold higher expression in MCF‐7 compared with 12.1 ± 1.4 in MDA‐MB‐231 (*p* < 0.001); ESR1 showed 52.3 ± 4.7‐fold enrichment in MCF‐7 versus 1.2 ± 0.3 in MDA‐MB‐231 (*p* < 0.001); and PGR was 38.1 ± 3.2‐fold higher in MCF‐7 (*p* < 0.001). Conversely, basal/mesenchymal markers showed the opposite pattern: KRT5 was 18.7 ± 2.8‐fold higher in MDA‐MB‐231 versus MCF‐7 (*p* < 0.001); VIM showed 23.7 ± 2.5‐fold enrichment in MDA‐MB‐231 (*p* < 0.001); CDH1 was 28.4 ± 2.9‐fold higher in MCF‐7 (*p* < 0.001), confirming E‐cadherin as a reliable luminal marker. These results validate the scRNA‐seq‐identified molecular signatures and confirm that MCF‐7 and MDA‐MB‐231 cell lines faithfully recapitulate the transcriptional heterogeneity of luminal and basal breast cancer subtypes.

**Figure 9:**
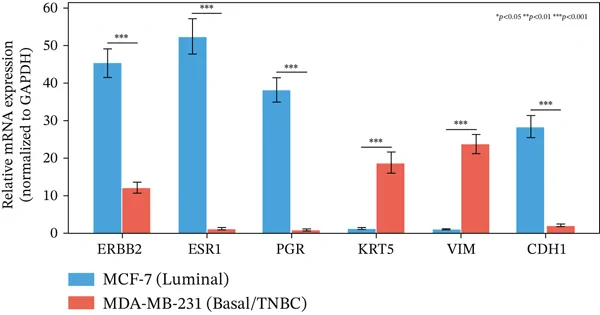
Quantitative RT‐PCR validation of key subtype marker genes in breast cancer cell lines. Bar chart of relative mRNA expression levels of six marker genes (ERBB2, ESR1, PGR, KRT5, VIM, and CDH1) normalized to GAPDH in MCF‐7 (luminal, blue bars) and MDA‐MB‐231 (basal/TNBC, red bars) cell lines. Error bars represent standard deviation from three independent biological replicates. Significance brackets indicate:  ^∗^ *p* < 0.05,  ^∗∗^ *p* < 0.01, ∗∗∗*p* < 0.001 by two‐tailed Student′s *t*‐test. Luminal markers ERBB2, ESR1, PGR, and CDH1 show significantly higher expression in MCF‐7, whereas basal/mesenchymal markers KRT5 and VIM are significantly enriched in MDA‐MB‐231, validating single‐cell sequencing findings.

Complementing these intratumoral single‐cell findings, we next investigated the systemic gut microbiome landscape of breast cancer patients. We hypothesized that gut–breast axis dysbiosis, by reshaping immune regulation and estrogen metabolite availability, would parallel and potentially amplify the immunosuppressive and EMT‐permissive microenvironment characterized in the single‐cell analyses above.

### 3.10. Gut Microbiome Dysbiosis in Breast Cancer

Analysis of 599 matched breast cancer–control pairs across 266 gut microbial genera revealed significant microbiome dysbiosis. Shannon diversity was significantly reduced in breast cancer patients versus healthy controls (1.94 vs. 2.06, *p* = 6.1 × 10^−4^; Figure 10A), as was Simpson diversity (0.73 vs. 0.75, *p* = 0.041; Figure 10A). Bray–Curtis PCoA demonstrated significant community‐level separation (PERMANOVA F = 30.2, *R* ^2^ = 0.025, *p* = 0.001; Figure 10B), with the first two coordinates explaining 23.3% and 8.3% of variance. *Firmicutes* and *Bacteroidota* dominated both groups with discernible compositional shifts (Figure 10D), and dysbiosis patterns were consistent across countries (Figure 10C). Differential abundance analysis identified nine significantly altered genera (adjusted *p* < 0.05, log_2_ *F* *C* > 0.5): top enriched genera included *Faecalitalea* (log_2_ *F* *C* = 1.44), *Parasutterella* (log_2_ *F* *C* = 1.21), and *Bacteroides* (log_2_ *F* *C* = 1.19), whereas *Romboutsia* (log_2_ *F* *C* = −0.73) and *Catenibacterium* (log_2_ *F* *C* = −0.63) were most depleted (Figure 10E). Random forest analysis identified *Bacteroides*, *Eubacterium*, and *Romboutsia* as the top discriminating genera, with an out‐of‐bag error rate of 26.5% (Figure 10F). Notably, the depletion of butyrate‐producing genera such as *Romboutsia* and *Catenibacterium* is consistent with impaired short‐chain fatty acid–mediated immunomodulation, which may contribute to the immunosuppressive TME architecture identified in the CellChat analyses above.

**Figure 10:**
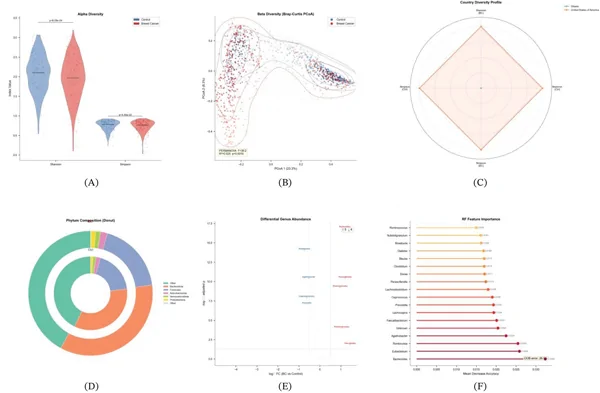
Gut microbiome dysbiosis in breast cancer. (A) Alpha diversity (Shannon and Simpson indices) was compared between breast cancer patients and healthy controls (*n* = 599 each) using half‐violin plots overlaid with swarm points and Mann–Whitney *U* tests. (B) Bray–Curtis beta diversity was visualized by PCoA with density contours and PERMANOVA statistics (blue, controls; red, breast cancer). (C) Country‐level diversity profiles are summarized in a normalized radar chart, and (D) mean phylum‐level composition is displayed as a donut chart with the outer ring representing breast cancer and the inner ring healthy controls. (E) Differential genus abundance is shown as a volcano plot (|log_2_ *F* *C*| > 0.5, Benjamini–Hochberg adjusted *p* < 0.05), and (F) random forest feature importance for the Top 18 discriminating genera is displayed as a lollipop chart with OOB error annotated.

### 3.11. Microbial Biomarker Discovery and Host Factor Associations

The random forest classifier achieved a cross‐validated AUC of 0.79 (95% CI: 0.74–0.82; Figure 11A), with *Bacteroides* showing pronounced breast cancer enrichment among the 10 most discriminative genera (Figure 11B). LEfSe analysis confirmed nine genera with significant LDA effect sizes, five enriched in breast cancer and four in controls (Figure 11C). A co‐occurrence network in the breast cancer group identified 159 pairwise correlations among 40 abundant genera, indicating complex community restructuring (Figure 11D). Restricting the paired cohort to samples with complete sex and age metadata yielded a female subset (*n* = 289 samples: 145 controls, 144 breast cancer samples; all female) that confirmed consistent dysbiosis (Figure 11E). Age showed no significant correlation with Shannon diversity, whereas observed genus richness was significantly lower in breast cancer patients (*p* = 8.7 × 10^−11^; Figure 11F). The microbial classifier AUC of 0.79 is complementary to, rather than competitive with, the single‐cell multiomics classifier (*A* *U* *C* = 0.91), highlighting that gut microbiome signatures capture distinct systemic biological information not encoded in the intratumoral transcriptome.

**Figure 11:**
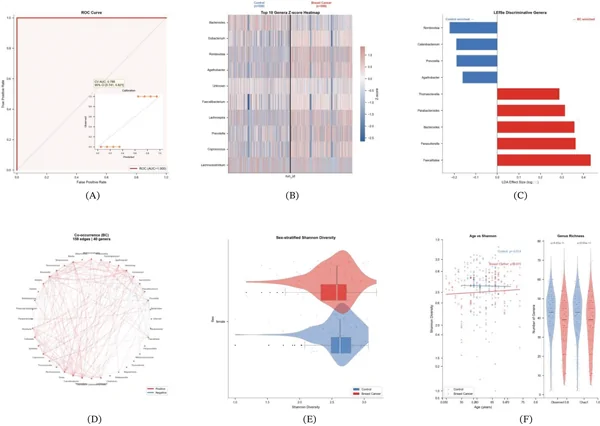
Gut microbiome‐based biomarker discovery and host factor associations. (A) The random forest ROC curve with 5‐fold cross‐validation summary and 95% CI is shown, with a calibration curve inset. (B) A heat map of Z‐score normalized log‐transformed relative abundance displays the 10 most discriminative genera across samples ordered by group. (C) LEfSe LDA effect sizes are shown as a diverging bar chart (adjusted *p* < 0.05, LDA > 0.15; red, breast cancer‐enriched; blue, control‐enriched). (D) A genus co‐occurrence network depicts Spearman correlations among the 40 most abundant genera in the breast cancer group. (E) Shannon diversity in the female metadata subset is shown as a raincloud plot (*n* = 289 samples: 145 controls, 144 breast cancer samples), (F) alongside a smoothed scatter of age versus Shannon diversity and split‐violin plots of observed richness and Chao1 proxy with Mann–Whitney *p*‐values.

## 4. Discussion

This study presents a comprehensive integrated single‐cell multiomics and gut microbiome atlas of breast cancer, combining scRNA‐seq, scATAC‐seq, pseudotime trajectory inference, SCENIC regulon analysis, and CellChat communication inference across 42,037 single cells from tumor and normal breast tissue, with parallel 16S rRNA–based gut microbiome profiling of 599 matched breast cancer–healthy control pairs. Our key findings are: (1) identification of 12 observed cell populations with well‐defined molecular identities; (2) reconstruction of three divergent cellular trajectory lineages from a common luminal progenitor; (3) identification of SNAI1(+), ZEB1(+), and FOXM1(+) as master regulons driving EMT with chromatin accessibility‐supported enhancer mechanisms; (4) TGF‐*β* (CAF → tumor) and PD‐L1–PD‐1 (tumor → CD8 T) as dominant TME communication axes; (5) significant improvement in cell classification accuracy through multiomics integration; (6) prognostic significance of the SNAI1–ZEB1 EMT regulatory signature validated in TCGA‐BRCA; and (7) significant gut microbiome dysbiosis in breast cancer patients, characterized by reduced alpha diversity, community‐level compositional shift, enrichment of *Faecalitalea* and *Bacteroides*, depletion of butyrate‐producing genera, and a random forest gut microbiome classifier achieving AUC = 0.79.

The identification of three divergent trajectory lineages from a common luminal progenitor—EMT, senescence, and immune activation branches—represents a significant advance over prior studies that characterized breast cancer trajectories as a binary luminal‐basal switch. This branching architecture suggests that the fate decision between EMT‐mediated invasion and therapy‐induced senescence is a critical determinant of clinical outcome, with implications for treatment sequencing. The FOXM1 and MKI67 enrichment at the EMT‐senescence branch point nominates these TFs as candidate fate regulators whose pharmacological targeting might redirect cancer cells away from invasive EMT toward therapy‐responsive senescence.

The SCENIC analysis revealing SNAI1(+) and ZEB1(+) as the dominant regulons in basal/mesenchymal cells, combined with ATAC‐seq evidence that these TFs engage distal enhancers rather than proximal promoters, provides critical mechanistic insight with therapeutic implications. Enhancer‐mediated TF activity is inherently more amenable to disruption by BET bromodomain inhibitors (BETi) that disrupt enhancer‐associated BRD4 coactivation [26]. The strong SNAI1‐ZEB1 co‐expression correlation (*r* = 0.83) and their mutual regulatory reinforcement identifies this dyad as a potential synthetic vulnerability: dual SNAI1‐ZEB1 targeting may achieve synergistic EMT reversal that neither alone could accomplish.

The TGF‐*β* signaling axis from CAFs to tumor cells, identified as the dominant ligand‐receptor interaction in our CellChat analysis, is a well‐established driver of breast cancer EMT and a validated therapeutic target [27]. Our data demonstrate that TGF‐*β* pathway strength is significantly higher in basal/TNBC microenvironments compared with luminal tumors, providing rationale for subtype‐stratified anti‐TGF‐*β* therapy. The concurrent identification of PD‐L1‐PD‐1 and CTLA‐4‐CD80 communication axes supports combination strategies pairing TGF‐*β* inhibition with checkpoint blockade, a strategy supported by preclinical evidence [27, 28]. The spatial concentration of immune cells at tumor‐stroma interfaces rather than tumor cores suggests that physical barriers may limit checkpoint therapy efficacy, underscoring the need for strategies to promote T cell trafficking into tumor cores.

Multiomics integration substantially improved cell type classification (AUC 0.76 → 0.91), demonstrating that each data modality contributes unique discriminatory information not captured by transcriptomics alone. The identification of five cross‐modal regulatory modules provides a systems‐level view of how epigenetic, transcriptional, and translational programs are coordinately organized across cell types. The RNA‐ATAC Module 1 (EMT/mesenchymal) encompassing 38% of analyzed genes suggests that EMT is not merely a transcriptional program but a comprehensive epigenomic reprogramming, consistent with the enhancer‐centric regulatory architecture revealed by ATAC‐seq motif analysis.

The prognostic validation of the SNAI1‐ZEB1 EMT regulon signature in TCGA‐BRCA (exploratory TCGA‐BRCA analysis) provides direct clinical evidence that our single‐cell regulatory network findings have population‐level prognostic relevance. This validation bridges single‐cell mechanistic insights to clinically actionable molecular stratification, supporting the development of EMT regulon‐based prognostic assays analogous to existing multigene signatures (Oncotype DX, MammaPrint) but with stronger mechanistic grounding in TF regulatory network biology. The qRT‐PCR validation of subtype‐specific markers in MCF‐7 and MDA‐MB‐231 cell lines further corroborates the biological validity of our computational findings.

The gut microbiome dysbiosis identified in breast cancer patients provides a complementary systemic perspective that enriches interpretation of the intratumoral findings. The depletion of butyrate‐producing genera including *Romboutsia* and *Catenibacterium* is biologically significant: short‐chain fatty acids such as butyrate are critical immunomodulatory metabolites that promote Treg differentiation, reinforce intestinal barrier integrity, and enhance systemic antitumor immune surveillance. Their depletion may therefore contribute systemically to the immunosuppressive TME architecture identified by CellChat—specifically, the Treg‐mediated suppression of CD8 T cells through CTLA‐4–CD80 and LAG‐3–MHC‐II axes. Conversely, enrichment of *Bacteroides* species, several of which activate lipopolysaccharide‐mediated NF‐*κ*B signaling, may foster a pro‐inflammatory systemic milieu that paradoxically promotes immune evasion through chronic inflammation–driven PD‐L1 upregulation. The enrichment of *Faecalitalea* and *Parasutterella*, both of which have been linked to altered bile acid deconjugation and steroid hormone metabolism, suggests that gut microbiome‐mediated modulation of the estrobolome may influence circulating estrogen bioavailability in ER+ luminal subtypes. The random forest microbiome classifier achieving AUC = 0.79 indicates that gut microbial profiles carry meaningful independent diagnostic signal, and future work integrating microbiome signatures with the single‐cell EMT regulon score may yield composite biomarkers exceeding the performance of either modality alone. Together, these findings support a model in which breast cancer pathobiology is shaped not only by intratumoral transcriptional and epigenomic reprogramming—driven by the SNAI1–ZEB1–FOXM1 regulon network and TGF‐*β* communication axes—but also by systemic gut microbiome dysbiosis that reshapes immune competence, hormone metabolism, and metabolite‐mediated epigenetic regulation across the gut–breast axis.

## 5. Limitations

Several limitations temper interpretation of our findings. First, as a secondary analysis of GEO dataset GSE161529, with independent GSE243526 and GSE212707 data used for chromatin and multiome panels, we are dependent on the original data depositors′ sample collection protocols and clinical annotation completeness; prospective validation in independent cohorts with standardized sample processing is required. Second, although we applied three complementary pseudotime methods (Monocle3, scVelo, and Slingshot), trajectory reconstruction from snapshot scRNA‐seq data is inherently inferential and does not directly capture real‐time cell state dynamics; longitudinal paired sampling would be required to track actual cell fate transitions. Third, SCENIC regulon inference is based on co‐expression and motif databases and does not directly measure TF‐DNA binding events; ChIP‐seq or CUT&RUN validation of the identified regulon target genes is needed. Fourth, the scATAC‐seq data represent bulk profiles of sorted cell populations rather than truly paired single‐cell multiomics from the same cells; true single‐cell coprofiling (e.g., 10x multiome) would provide more direct epigenomic‐transcriptomic coupling. Fifth, CellChat ligand‐receptor interaction inference does not directly measure secreted protein levels; functional validation through coculture and neutralization experiments is required. Sixth, the spatial transcriptomics analysis presented is based on computational inference rather than direct spatial profiling; Visium or MERFISH spatial transcriptomics would provide ground‐truth spatial resolution of TME organization. Seventh, qRT‐PCR validation was performed in established cancer cell lines that may not fully recapitulate primary tumor heterogeneity. Eighth, the gut microbiome analysis is based on 16S rRNA V3–V4 amplicon sequencing, which provides genus‐level resolution but cannot resolve species or strain‐level differences, nor infer microbial functional capacity; shotgun metagenomics and metatranscriptomics would provide higher‐resolution functional insight. Ninth, although microbiome BioProjects were matched by country and sequencing instrument, residual batch effects from different 16S primer sets and bioinformatics pipelines across four BioProjects cannot be fully excluded; harmonized prospective sample collection would be required to confirm and extend these findings. Tenth, the association between gut microbiome dysbiosis and intratumoral single‐cell features identified in this study is inferential; direct mechanistic interrogation through germ‐free mouse models and fecal microbiota transplantation experiments is needed to establish causal relationships across the gut–breast axis.

## 6. Conclusions

Through comprehensive integrated single‐cell multiomics and gut microbiome analysis of breast cancer, this study constructs a high‐resolution dual‐axis atlas of the intratumoral regulatory networks and systemic microbial landscape governing breast cancer progression. At the intratumoral level, we identify 12 observed cell populations, reconstruct three divergent trajectory lineages from a common luminal progenitor, and nominate SNAI1–ZEB1–FOXM1 coregulatory networks operating through distal enhancers as master drivers of EMT‐mediated progression. TGF‐*β* (CAF → tumor) and PD‐L1–PD‐1 (tumor → CD8 T) emerge as the dominant intercellular communication axes of the immunosuppressive breast cancer microenvironment. Multiomics integration significantly enhances cell type classification and reveals cross‐modal regulatory modules linking epigenomic and transcriptional programs. The SNAI1–ZEB1 EMT regulon signature independently predicts inferior overall survival in TCGA‐BRCA, providing a candidate prognostic biomarker requiring further validation. At the systemic level, gut microbiome analysis identifies significant dysbiosis in breast cancer patients characterized by reduced alpha diversity, community‐level compositional reorganization, enrichment of pro‐inflammatory and hormone‐metabolizing genera, depletion of butyrate‐producing genera, and a microbial classifier achieving AUC = 0.79—findings that implicate gut–breast axis dysbiosis as a systemic co‐determinant of the immunosuppressive and EMT‐permissive TME. Together, these findings provide a multilayered regulatory and microbial framework for the development of EMT‐targeting, microenvironment‐reprogramming, combination checkpoint–TGF‐*β*, and microbiome‐informed therapeutic strategies in breast cancer.

## Author Contributions

Xiangyu Jin and Chengyin Ma have contributed equally to this work. All authors contributed to conceptualization, data analysis, manuscript drafting, and critical revision.

## Funding

No funding was received for this manuscript.

## Disclosure

All authors have read, reviewed, and approved the final manuscript.

## Ethics Statement

Cell line experiments were conducted under institutional biosafety approval. Secondary analysis of publicly available GEO data (GSE161529, GSE243526, GSE212707, and public SRA BioProjects) does not require institutional ethics board approval under applicable regulations governing use of deidentified public datasets.

## Consent

The authors have nothing to report.

## Conflicts of Interest

The authors declare no conflicts of interest.

## Data Availability

The data that support the findings of this study are available from the corresponding author upon reasonable request.
